# PLA2G6 gene mutation and infantile neuroaxonal degeneration; report of three cases from Iran

**DOI:** 10.22038/ijbms.2021.55082.12340

**Published:** 2021-09

**Authors:** Reza Jafarzadeh Esfehani, Atieh Eslahi, Mehran Beiraghi Toosi, Ariane Sadr-Nabavi, Mohammad Amin Kerachian, Mahsa Sadat Asl Mohajeri, Mahsa Farjami, Farzaneh Alizade, Majid Mojarrad

**Affiliations:** 1 Medical Genetics Research Center, Mashhad University of Medical Sciences, Mashhad, Iran; 2 Department of Medical Genetics, Faculty of Medicine, Mashhad University of Medical Sciences, Mashhad, Iran; 3 Student Research Committee, Mashhad University of Medical Sciences, Mashhad, Iran; 4 Department of Paediatric Neurology, Ghaem Medical Centre, Faculty of Medicine, Mashhad University of Medical Sciences, Mashhad; 5 Iranian Academic Center for Education, Culture and Research, (ACECR), Mashhad, Iran

**Keywords:** Developmental disabilities Magnetic resonance imaging, Neuroaxonal dystrophies, Pantothenate kinase- associated neuro- degeneration, Whole exome sequencing

## Abstract

**Objective(s):**

Infantile neuroaxonal degeneration (INAD) is a rare subgroup of neurodegeneration with brain iron accumulation (NBIA) disorders. This progressive disorder may develop during the early years of life. Affected individuals mostly manifest developmental delay and/or psychomotor regression as well as other neurological deficits. In the present study, we discussed 3 INAD patients diagnosed before the age of 10 by using Whole-Exome Sequencing (WES).

**Materials and Methods:**

We evaluated 3 pediatric patients with clinical phenotypes of INAD who underwent WES. Sanger sequencing was performed for co-segregation analysis of the variants in the families. An *in-silico *study was conducted for identification of the molecular function of the identified genetic variants in the *PLA2G6* gene.

**Results:**

We detected three novel genetic variants in the PLA2G6 gene including a homozygous missense (NM_003560.2; c.1949T>C; p.Phe650Ser), a splicing (NM_001349864; c.1266-1G>A) and a frameshift variant (NM_003560.4; c.1547_1548dupCG; p.Gly517ArgfsTer29). Since the variants were not previously reported in literature or population databases, we performed in-silico studies for these variants and demonstrated their potential pathogenicity.

**Conclusion:**

The current study reports novel genetic variants in the *PLA2G6* gene in the Iranian population, emphasizing the importance of high-throughput genetic testing in rare diseases.

## Introduction

**Figure 1:**
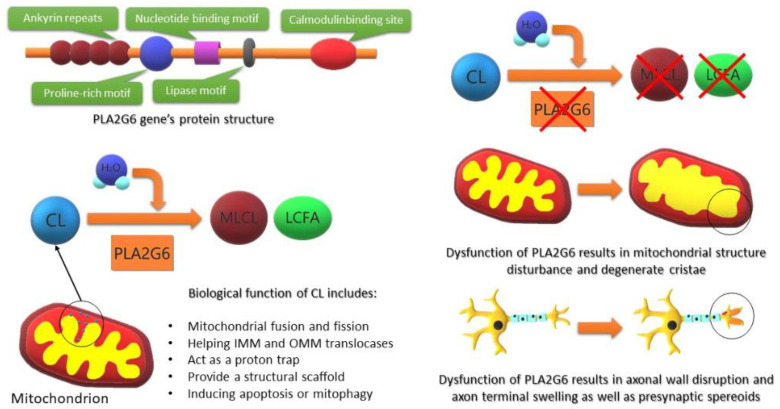
Schematic effect of PLA2G6 gene structure and main effects on its mutation effect on normal cellular function

Neurodegeneration with brain iron accumulation (NBIA) is a group of heterogeneous disorders that are rarely diagnosed in neurology clinics. NBIA disorders share a common brain finding which is iron accommodation in specific brain regions. However, the exact mechanism of iron accommodation is not clearly understood (1). Infantile neuroaxonal degeneration (INAD) is a subgroup of NBIA and a very rare neurological disease with an unknown prevalence stated as an “ultra-rare” disorder (2). INAD usually affects children younger than 3 years of age and manifests by developmental delay and progressive neurologic deficits. The main gene involved in this genetic disease is the *PLA2G6*, encoding calcium-independent phospholipase A2 group VIa protein. The *PLA2G6* gene product plays an important role in phospholipids’ metabolism (1, 3) (Figure 1). The diagnosis of this rare syndrome mainly depends on clinical examinations as well as brain imaging studies. However, according to the recent diagnostic genetic tests, the diagnosis of such a rare syndrome is growing. In the present report, we discussed the first cases of INAD in Iran diagnosed by next-generation sequencing (NGS).

## Materials and Methods

Three pediatric patients who were referred to the Pediatric Neurogenetic Clinic of Ghaem Hospital were included in the present case study. The patients’ parents gave informed consent for participating in our study and the study was conducted according to the Helsinki Declaration and also approved by the department of medical genetics institutional review board (Mashhad University of Medical Sciences, Mashhad, Iran). Every patient underwent neurologic examination by a pediatric neurologist and a full family pedigree was drawn. Five milliliters of peripheral blood were taken from each patient and the extraction of genomic DNA was performed by DNA purification kit (Roche, Switzerland) according to the manufacturer’s instructions and the quantity and quality of the extracted genomic DNA was evaluated using a nanodrop instrument (Thermo Fisher Scientific, USA). The samples were sent for WES by Illumina Sequencer (Illumina, San Diego, CA, USA).

## Results

***First case***

**Figure 2:**
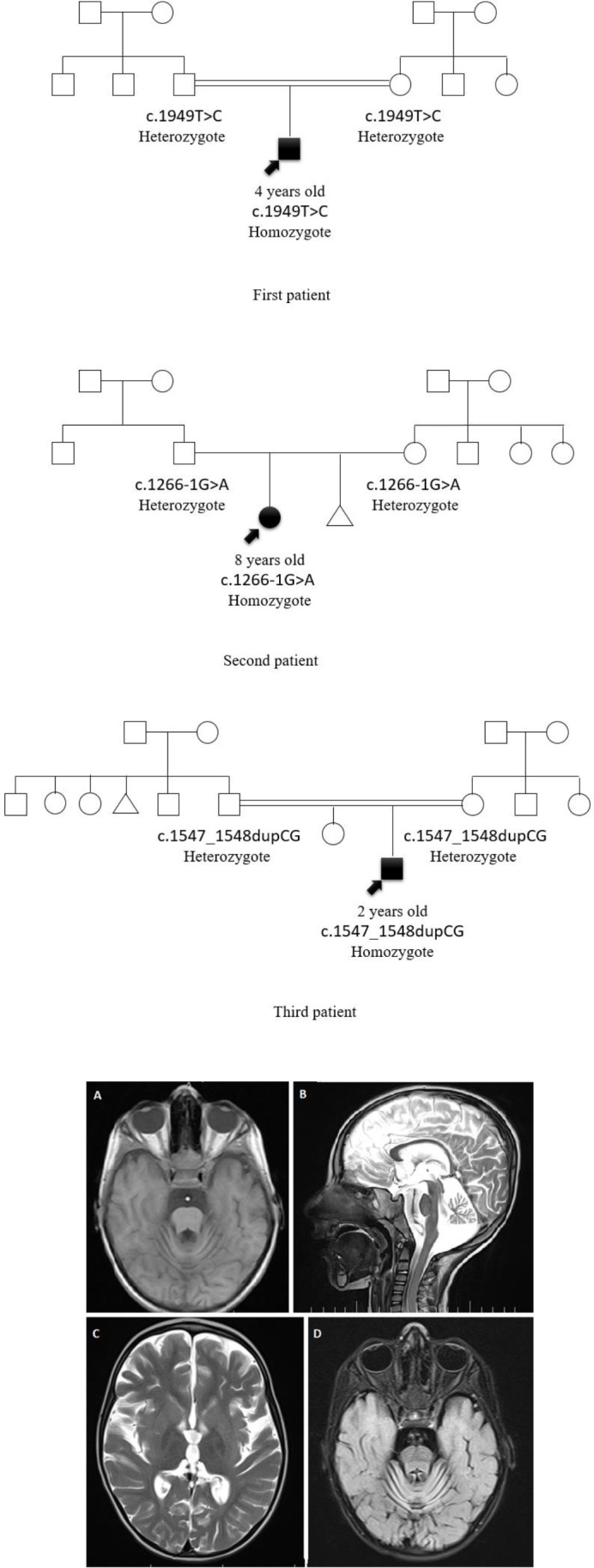
Patient pedigree and magnetic resonance imaging of the brain in T1 (A), sagittal T2 (B), axial T2 (C), and FLAIR (D) section demonstrating cerebellar atrophy (B)

A 4 years old boy was referred to the pediatric neurology outpatient clinic because of delayed motor and intellectual development (Table 1). The patient was the first child of a consanguineous family and could sit independently from 8 months of age and say only one word. However, the sitting and verbal ability regressed and the spastic gate developed gradually. By age 2, the patient developed episodes of seizures and gradual signs of hearing difficulties as well as visual loss. Electroencephalography (EEG) revealed sharp and spike waves but the brain magnetic resonance imaging (MRI) was unremarkable. ABR (auditory brainstem response) and cortical auditory evoked potentials revealed hearing difficulties in the left ear and ocular examination revealed bilateral optic disk atrophy. Although antiepileptic drugs were started for the patient, the family did not complete the follow-up visits. As the symptoms worsened, the patient was referred at the age of 4. At this age, he was not able to sit independently and talk. Moreover, he could not keep his head straight when siting or lying down. At the time of physical examination, nystagmus was obvious. Truncal hypotonia, as well as mild brisk deep tendon reflexes, were prominent. According to the progressive course of the disease and the presence of nystagmus, in addition to visual problems and gross motor skill regression, another brain MRI, electromyography, and a nerve conduction study (EMG-NCS) were ordered. MRI indicated cerebellar atrophy and deposition of metal in Globus pallidus (Figure 2). Also, EMG-NCS revealed evidence of ongoing axonal loss. 

***Next-generation sequencing and bioinformatics analysis***

**Figure 3:**
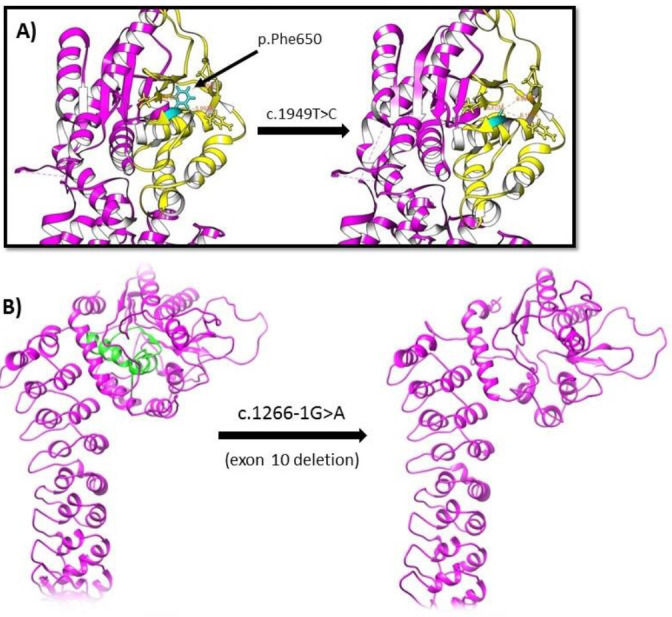
Comparative molecular model of the wild-type and mutant iPLA2. (A) Model representation of the c.1266-1G>A splicing mutation. The corresponding deleted section of the mutant protein that is related to exon 10 is depicted as green in the wild-type left side model. (B) Position of iPLA2 p.Phe650Ser mutation is highlighted in cyan and the active site region is in yellow. The left model represents the normal protein and the right side shows the mutated protein

Due to the disease’s progressive course and clinical symptoms including both visual and auditory problems along with seizures and brain MRI findings, indicating cerebellar atrophy and evidence of brain deposits, we decided to perform a whole-exome sequencing (WES) study to confirm the diagnosis of INAD. The extracted variants were annotated and coding region variants were filtered based on allele frequency (mean allele frequency < 3%), disease-causing variants (frameshifts, splice site, missense and stop gain/loss variants), and zygosity status (homozygous variants). The filtration resulted in 1205 variants and these variants were evaluated based on their effect on neuronal development pathways and similarity of abnormal phenotypes of the patient and phenotypes provided by the OMIM database for each variant. Among these variants, a missense variant in *PLA2G6* gene (NM_003560.2, chr22:38,511,619 c.1949T>C, p.Phe650Ser) was found. The mutation was a novel missense mutation and was present in neither 1000G nor ExAC nor Iranome population-based databases. The p.Phe650Ser was a pathogenic variant based on various prediction tools including BayesDel_addAF, DANN, DEOGEN2, EIGEN, FATHMM-MKL, LIST-S2, M-CAP, MVP, MutationAssessor, MutationTaster, and SIFT. Moreover, it was predicted as a pathogenic variant based on the American College of Medical Genetics 2015 criteria for pathogenicity of a genetic variant. Modeling of the iPLA2 F650S mutation was performed using Chimera software based on the known structure of the iPLA2 protein (Protein Data Bank (PDB) 6AUN) (4). In the model of p.Phe650Ser mutation, the altered serine amino acid due to the smaller size of the side-chain leads to perturbation of the secondary structure of the protein (Figure 3A). From these findings, we considered this variant to be a pathogenic mutation. 

***Second case***

The second patient was an 8-year-old girl with the same disease course as the first patient (Table 1). The disease has a progressive nature and the patient had signs of developmental delay and intellectual disability. The patient developed seizures and hearing difficulties as well as nystagmus with visual impairment. With the suspicion of organic acidemias early in her life, the coding regions and splicing sites of *GCDH, ETFA, ETFB, ETFDH, IVD *were evaluated by PCR and sequencing and no pathogenic variant was identified. The clinical phenotype was also suggestive of Rett syndrome and the results of *MECP2* sequencing were unremarkable. 

***Next-generation sequencing and bioinformatic analysis***

According to the suggestive phenotype of INAD, the patient underwent WES. The extracted variants were annotated and coding region variants were filtered similar to the first patient. Among 13568 variants, a splicing variant in the *PLA2G6* gene (NM_001349864, chr22: 38519266, c.1266-1G>A) was reported. This variant was a novel splicing mutation. The variant was neither found in 1000G nor ExAC nor Iranome population databases. There were pathogenic predictions from BayesDel_addAF, DANN, EIGEN, FATHMM-MKL, MutationTaster, and scSNV-Splicing prediction tools. It was predicted as a pathogenic variant based on the American College of Medical Genetics 2015 criteria for pathogenicity of a genetic variant. The splicing mutation model was obtained from the I-TASSER server using the FASTA protein sequence (NM_001349865)(5). The effect of this mutation was analyzed with Chimera modeling software (6). In the molecular model of c.1266-1G>A splicing mutation, the corresponding region to the exon has been deleted, depicted in green, containing the oxyanion hole and a part of the active site (Figure 3B)(4). Thus, the impact of this splicing mutation on the protein function is severe and we have considered this variant a pathogenic mutation since this mutation leads to skipping of exon 10. 

***Third case***

A 2 years old boy with psychomotor regression was asymptomatic until 8 months of age and then gradually developed hypotonia and poor feeding as well as reduced deep tendon reflexes (Table 1). The child underwent EMG to investigate the possible neurologic causes. The results indicated a chronic motor neuropathy/axonopathy, and a genetic diagnostic workup for spinal muscular atrophy was ordered. The molecular genetic analysis of the *SMN1* gene did not reveal any deletion. As the child developed hearing and visual impairment, a brain MRI, an auditory brain response test, and electroretinography were ordered. The MRI was not remarkable while bilateral hearing impairment and cone rode dystrophy were reported in the visual and hearing examinations. So, with a primary diagnosis of INAD, the patient underwent WES. 

***Next-generation sequencing and bioinformatic analysis***

Due to the possible clinical diagnosis of INAD, the patient underwent WES similar to other patients. The genetic variants were annotated and coding region variants were filtered. Among 11265 variants, a frameshift variant in the 11th exon of the *PLA2G6* gene (NM_003560.4, Chr12:38519145, c.1547_1548dupCG, p.Gly517ArgfsTer29) was identified. The variant was not previously reported in the literature and its frequency was unknown. Moreover, the variant was pathogenic based on both the phyloP prediction tool and the American College of Medical Genetics 2015 criteria for pathogenicity of a genetic variant. The segregation study revealed that the parents were heterozygote for this damaging mutation. The patient’s parents decided to have a child after the determination of their child’s genotype. The prenatal diagnosis for p.Gly517ArgfsTer29 mutation in the *PLA2G6* gene revealed that the fetus was a normal homozygote for the mutation and the mother gave birth to a healthy child. 

**Table 1:** Demographic and genetic information of the 3 patients with infantile neuroaxonal dystrophy

**Clinical findings**										
	**Age**	**Gender**		**Chief compliant**		**Symptoms**	**EEG**	**Ocular examination**	**MRI**	**EMG-NCS**
**First patient**	4 years old	Male		Delayed motor and intellectual development		Seizures; hearing difficulties; visual loss and nystagmus; truncal hypotonia; brisk deep tendon reflex	Sharp and spike waves	Bilateral optic disk atrophy	Cerebellar atrophy and deposition of metal in Globus pallidus	ongoing axonal loss
**Second patient**	8 years old	Female		Delayed motor and intellectual development		Seizures; hearing difficulties; visual loss and nystagmus; brisk deep tendon reflex	Sharp and spike waves	Not performed	Cerebellar atrophy	Not performed
**Third patient**	2 years old	Male		Delayed motor and hypotonia		Hearing impairment; cone-rod dystrophy; hypotonia and reduced deep tendon reflexes	-	Cone rod dystrophy	Normal	Chronic motor neuropathy
**Genetic, bioinformatics, and molecular studies**										
	**WES Variants in ** ***PLA2G6*** ** gene**	**Frequency in 1000 Genome, ExAC, and Iranome databases **	**Sanger sequencing **		** Mutation taster**	**SIFT and PolyPhen**	**Chimera protein modeling software (based on Protein Data Bank (PDB) 6AUN)**			
**First patient**	(NM_003560.2) chr22:38,511,619 c.1949T>C p.Phe650Ser	Not reported	Confirmed		Disease causing mutation	Deleterious	Altered serine amino acid due to the smaller size of the side chain, leads to perturbation of the secondary structure of the protein.			
**Second patient**	(NM_001349864) chr22: 38519266 c.1266-1G>A	Not reported	Confirmed		Disease-causing mutation	Deleterious	Impact of this splicing mutation on the protein function is so severe and this variant may be considered as a pathogenic mutation since this mutation leads to skipping of exon 10.			
**Third patient**	(NM_003560.4) Chr12:38519145 c.1547_1548dupCG p.Gly517ArgfsTer29	Not reported	Confirmed		Disease-causing mutation	Deleterious	Frame-shift variant at the exome 11 will have a great impact on PLA2G6 protein.			

## Discussion

In the present case series, we have discussed the first cases of a rare form of Phospholipase A2-associated neurodegeneration (PLAN) in Iran. PLAN is a subgroup of NBIA which is mainly inherited in an autosomal recessive manner (7). INAD, which is also called a classical PLAN, develops in early life and progresses to a debilitating disorder (7). The same as our patients, affected children are usually normal at birth without any apparent dysmorphic feature. Hypotonia and psychomotor regression usually develop before age 3 and spastic features could develop in later life (8). Some clinical and imaging features are characteristics in INAD patients. Nystagmus and pallor optic disk are 2 main ophthalmic findings that are reported in INAD patients. Also, optic nerve degeneration may be prominent in brain imaging studies (9, 10). Our third case developed cone rode dystrophy which is a very rare finding in INAD patients. Other neurologic manifestations which are not common in the early stages are seizures and abnormal EEG patterns. One of our patients developed seizures from age 3 with predominant sharp and spike waves while the other two did not have any sign of seizures. Other EEG findings including frontal-predominant fast rhythms have also been reported in INAD patients (2). Cerebellar atrophy in brain MRI is a prominent finding in patients. Another major finding is a sign of iron deposit in the brain which is mostly seen around globus pallidus (11). Our present study revealed that the development of brain pathologies could be age-dependent in INAD patients as our third patient did not develop any abnormal signs in brain imaging. Our third patient did not develop any of these two brain pathologies in his brain MRI. Similar to our results, Romani *et al*. also demonstrated that the development of cerebellar atrophy which is an important diagnostic clue for INAD may not be presented before age 2 (12). They reported that almost all of their patients with infantile and childhood-onset PLAN have hypertrophy of the Clava even as the only brain MRI finding. The presence of the specific clinical findings accompanied by brain imaging findings in a child who is suffering from developmental delay or psychomotor regression could be enough to clinically establish the diagnosis of INAD followed by ordering further confirmatory tests (13). 

Nowadays, according to recent development in genetic techniques, NGS techniques have become a cornerstone for confirmation of rare genetic disorders. In our patients, we decided to confirm our diagnosis by conducting WES. According to the WES results, two mutations have been detected in the *PLA2G6* gene. The three-dimensional models of the wild-type and mutant iPLA2 were used to determine the adverse effects of the mutations on the protein structure. Comparison of the models indicates both p.Phe650Ser and c.1266-1G>A mutations affect the protein active site, subsequently, impair protein-ligand interaction and phospholipase activity of the protein (Figure 1). 

*PLA2G6* gene encodes a calcium-independent group VIA phospholipase A2 enzyme(3). Although the exact role of mutation in this gene and developing the symptoms of INAD is not widely understood, there could be some possible explanations. The main role of the *PLA2G6* product is to maintain the hemostasis of the cellular membrane. Mutations in this gene can affect neuronal cells as well as mitochondrial wall membrane dynamics. Within the inner membrane of the mitochondrial membrane, calcium-independent *PLA2G6* hydrolyzes, form monolysocardiolipin (MLCL) from cardiolipin (CL) by removing acyl chains. Mutation in *PLA2G6* could disrupt this process, resulting in mitochondrial dysfunction and disruption of the axonal wall (Figure 1) (14). Furthermore, induced calcium dysregulation has been reported to be another cause of INAD symptoms. Illingworth *et al*. reported 5 cases of INAD and addressed mitochondrial changes in one of their patients. They have performed muscle biopsy and reported neurogenic changes as well as reduced cytochrome oxidase activity on respiratory chain analysis. Such findings indicate that the effect of oxidative stress along with calcium dysregulation could further degenerate the mitochondrial function of neuronal cells (15). 

Taking all together, our study demonstrates that according to the variable manifestations of INAD, diagnosis of this rare neurological disease requires a high clinical suspicion. As mentioned earlier, our cases were first presented with unspecific clinical features mimicking other neurological diseases including SMA and Rett syndrome. A more common differential diagnosis of INAD is ceroid lipofuscinosis. Ceroidolipofuscinosis can be excluded by the absence of seizures and myoclonus with normal EEG and cortical atrophy on MRI. Moreover, disorders of glycosylation sharing cerebellar atrophy should always be considered and can easily be ruled out by performing biochemical tests (14).

## Conclusion

INAD is a rare progressive neurogenetic disorder that is mostly diagnosed in early childhood. We have reported 3 genetic alterations in the Iranian patients in the *PLA2G6* gene with a suggestive phenotype of INAD by using WES. According to the rare nature of this syndrome and a broad range of differential diagnoses, pediatricians should be aware of this syndrome and consider it in every child presented with psychomotor regression even with normal imaging findings. 

## Authors’ Contributions

MM Study conception and design; RJ, AE, MS AM, MF Data analyzing and draft manuscript preparation; RJ, AE, MAK, ASN Data Processing, Collection, Perform Experiment; MM Critical revision of the paper; MM Supervision of the research; RJ, AE, MBT, ASN, MAK, MSAM, MF, FA, and MM Final approval of the version to be published (the names of all authors must be listed). 

## Compliance with Ethical Standars

Informed consent was obtained from all participating subjects according to the Declaration of Helsinki.

## Conflicts of Interest

The authors do not have any conflict of intrest to declare. 
